# Lung Involvement Patterns in COVID-19: CT Scan Insights and Prognostic Implications From a Tertiary Care Center in Southern India

**DOI:** 10.7759/cureus.53335

**Published:** 2024-01-31

**Authors:** Suhasini Balasubramaniam, Bharathi Priya Raju, Sowmya Perumpallipatty Kumarasamy, Swaminathan Ramasubramanian, Amitesh Krishna Srinivasan, Ishwar Gopinath, Kamakshi Shanmugam, Aravind S Kumar, Varun Visakan Sivasakthi, Srinidhi Srinivasan

**Affiliations:** 1 Radiodiagnosis, Government Stanley Medical College and Hospital, Chennai, IND; 2 Radiodiagnosis, Jawaharlal Institute of Postgraduate Medical Education and Research, Puducherry, IND; 3 Radiodiagnosis, Government Medical College, Omandurar Government Estate, Chennai, IND; 4 Internal Medicine, Government Medical College, Omandurar Government Estate, Chennai, IND; 5 Orthopaedics, Kovai Medical Centre and Hospital Institute of Health Sciences and Research, Coimbatore, IND; 6 Radiodiagnosis, Alluri Sitarama Raju Academy of Medical Sciences College and Hospital, Eluru, IND

**Keywords:** lung involvement, patterns, distribution, computed tomography, covid-19

## Abstract

Background

COVID-19, caused by the SARS-CoV-2 virus, has presented an unparalleled challenge and a profound learning curve globally. Among the myriad of investigative tools, CT scans of the chest have become instrumental in assessing the magnitude of lung involvement in the pathogenesis of this disease.

Objectives

This study aimed to evaluate the distribution and patterns of lung involvement depicted in the CT chest scans of COVID-19 patients admitted to a specialized tertiary care center located in a southern state of India.

Methods

With clearance secured from the Institutional Ethics Committee, an analytical cross-sectional study was conducted. It encompassed CT chest images from all symptomatic COVID-19 patients within the designated study center during the specified study timeline. Subsequent data analysis ensued.

Results

Among the 1066 COVID-19 patients evaluated, ground-glass opacities (GGO) were the predominant lung involvement pattern. Distinct patterns, such as GGOs combined with solid consolidation or atelectasis, were noted, with the highest mortality linked to GGOs paired with pneumomediastinum (PM). Data underscored a direct correlation between the extent of lung involvement and patient prognosis, with specific lung regions, namely the right apical, right posterior, right superior basal, left superior lingular, and left inferior lingular segments, showing frequent involvement.

Conclusion

Amidst the pandemic, our study emphasizes that ground-glass opacities on CT scans are robust indicators of COVID-19 in RT-PCR-positive patients. Early identification can enhance patient management, with findings highlighting a strong link between lung involvement and prognosis. This insight aids in refining patient triage, while further research is warranted to delve deeper into variations in lung involvement and guide treatment advancements.

## Introduction

The emergence of the SARS-CoV-2 virus, commonly referred to as the coronavirus, has precipitated the global crisis of the coronavirus disease-19 (COVID-19) pandemic [1]. Initially identified in Wuhan, China, in 2019, this pathogen has witnessed an exponential spread across international borders, instigating significant apprehensions [2]. Transmission predominantly occurs through respiratory droplets during human-to-human interactions, manifesting in a range of symptoms, notably myalgia, dyspnea, cough, and fever [3].

Prompt diagnosis and subsequent isolation of affected individuals remain paramount in curtailing the virus' dissemination. In the realm of radiology, CT scans have increasingly become the diagnostic modality of choice for suspected COVID-19 cases [4]. Their ability to identify ground-glass opacities (GGO), a recurrent radiological feature in the lungs of COVID-19 patients, offers distinct advantages [5]. Notably, CT scans boast a rapid turn-around time in stark contrast to RT-PCR tests, thereby expediting patient diagnosis [6].

This cross-sectional study aims to elucidate the morphological distribution and patterns of lung involvement in COVID-19 patients, as evidenced by chest CT scans. A comprehensive understanding of these patterns and their potential correlation with mortality may pave the way for refined diagnostic and therapeutic strategies. Amidst the tumultuous backdrop of the ongoing pandemic, enhancing our capability to swiftly gauge lung involvement and allocate patients accordingly is of paramount importance, ensuring an optimized and rational approach to patient care.

## Materials and methods

Study design and setting

This study was carried out as a retrospective cross-sectional investigation based in a hospital setting, with a primary focus on the analysis of CT chest images. Institutional Ethics Committee approval was granted under the reference IEC No. 24/IEC/GOMC/2021. The research adhered to the ethical guidelines set forth by the institutional research committee and complied with the principles outlined in the 1964 Helsinki Declaration, including its subsequent amendments or equivalent ethical standards. The study was conducted at the Department of Radiodiagnosis, Government Medical College, Omandurar Government Estate, Chennai, which was designated as the COVID-19 Care Center during the start of the pandemic.

Study period

The data collection spanned from April to June 2021, a period characterized by a significant surge in COVID-19 cases.

Participants

The research cohort consisted of adult individuals, aged 18 years and older, who were hospitalized within the specified study timeframe. Informed consent was procured via telephonic interviews using contact information available in the medical records.

Inclusion criteria

The inclusion criteria of this study encompassed various factors aimed at identifying eligible patients for participation. Patients meeting the following conditions were considered for inclusion in this investigation.

Participants were required to be 18 years of age or older upon admission to the healthcare facility. Additionally, candidates were expected to exhibit clinical symptoms consistent with COVID-19, which included the presence of fever or chills, coughing, shortness of breath or difficulty breathing, fatigue, muscle or body aches, headache, new loss of taste or smell, sore throat, congestion or runny nose, nausea or vomiting, and diarrhea [7]. Furthermore, a critical criterion for inclusion necessitated a confirmed positive test result for COVID-19 during their hospital stay.

These comprehensive criteria were meticulously established to ensure the selection of individuals who met the specified conditions for the study.

Exclusion criteria

Participants were excluded if they were under 18 years of age, if they did not exhibit clinical symptoms consistent with COVID-19 [7], or if they did not have a confirmed positive RT-PCR test for COVID-19.

Sample size

From the eligible cohort of patients fitting these criteria, 1,066 patients were selected based on convenience sampling.

CT chest imaging protocol

A non-contrast-enhanced chest CT scan was performed in the supine position using a 16-section multidetector CT scanner (Aquilion Lightning model TSX 035a, Toshiba), with patients maintaining a single inspiratory breath hold. Image analysis was conducted utilizing Vitrea software version 6.5.99 (Vital Images, Inc., Ootawara, Tochigi), adhering to specific parameters: window width set between 1000 and 2000 Hounsfield units (HU) and window level adjusted from −700 to −500 HU. The examination encompassed the entire lung region, extending from the apex to the costophrenic angles. Interpretation of the CT images was initially performed by two radiologists possessing a cumulative 15 years of experience. Subsequently, a reassessment was undertaken collaboratively with residents and interns, focusing on three anatomical landmarks: the apex, hilum, and base. A double-blinded method was employed for the distribution of images for review. Any interpretative disagreements were adjudicated by a senior radiologist with 20 years of experience.

Quantification of pulmonary involvement

For the assessment of lung involvement, the pulmonary architecture was segmented into 20 bronchopulmonary sections. The degree of opacification in each segment was meticulously evaluated. A scoring system was devised wherein each segment was assigned a maximum score of two, indicative of opacification affecting over half of the segment. Segments with less than half involvement received a score of one. This scoring mechanism enabled a comprehensive assessment, with a total maximum score of 40, where each unit score represented 2.5% of the affected lung parenchyma. This scoring system is shown in Tables 1-2.

**Table 1:** Basis of computed tomography severity scoring (CTSS)

Involvement of bronchopulmonary segment by visual assessment	Assigned score
Nil	0
Less than 50%	1
More than 50%	2

**Table 2:** Scoring sheet for reporting lung involvement Visual assessment score is based on Table 1

Segment No.	Segment name	Lung	Visual assessment score (0-2 per segment)
1	Left apical	Left	
2	Left posterior (assumed)	Left	
3	Left anterior	Left	
4	Left superior lingular	Left	
5	Left inferior lingular	Left	
6	Left superior basal	Left	
7	Left medial basal (assumed)	Left	
8	Left lateral basal	Left	
9	Left posterior basal	Left	
10	Left anterior basal	Left	
11	Right apical	Right	
12	Right anterior	Right	
13	Right posterior	Right	
14	Right medial	Right	
15	Right lateral	Right	
16	Right superior basal	Right	
17	Right anterior basal	Right	
18	Right medial basal	Right	
19	Right lateral basal	Right	
20	Right posterior basal	Right	

Data management

Information extracted from the CT images and patient records was systematically entered into a Microsoft Excel spreadsheet (Microsoft® Corp., Redmond, WA). This data entry process adhered to strict confidentiality and privacy protocols.

Statistical analysis

Descriptive statistics was employed to analyze the data using Microsoft Excel. The primary objective was to identify prevalent patterns and trends within the dataset, particularly focusing on lung involvement and its potential correlation with broader clinical presentations. The results were organized in tabular form to facilitate both comparative and inferential analyses.

Ethical considerations

The study was conducted adhering to the ethical guidelines of our institution, ensuring the confidentiality and anonymity of patient data. To ensure anonymity, patient identification information was removed from the data using the PACS software. Unique random numbers were then assigned to each image and clinical data record to allow linking while maintaining anonymity. Data were securely stored in the computer system of the Department of Radiodiagnosis in a password-protected folder. All procedures performed in studies involving human participants were in accordance with the ethical standards of the institutional and/or national research committee.

## Results

Our comprehensive analysis spanned 1066 patients, ranging in age from 18 to 92 years, with a mean age of 49.38 years (±14.85 standard deviations). The cohort included 580 men and 486 women.

CT chest image patterns

**Figure 1:**
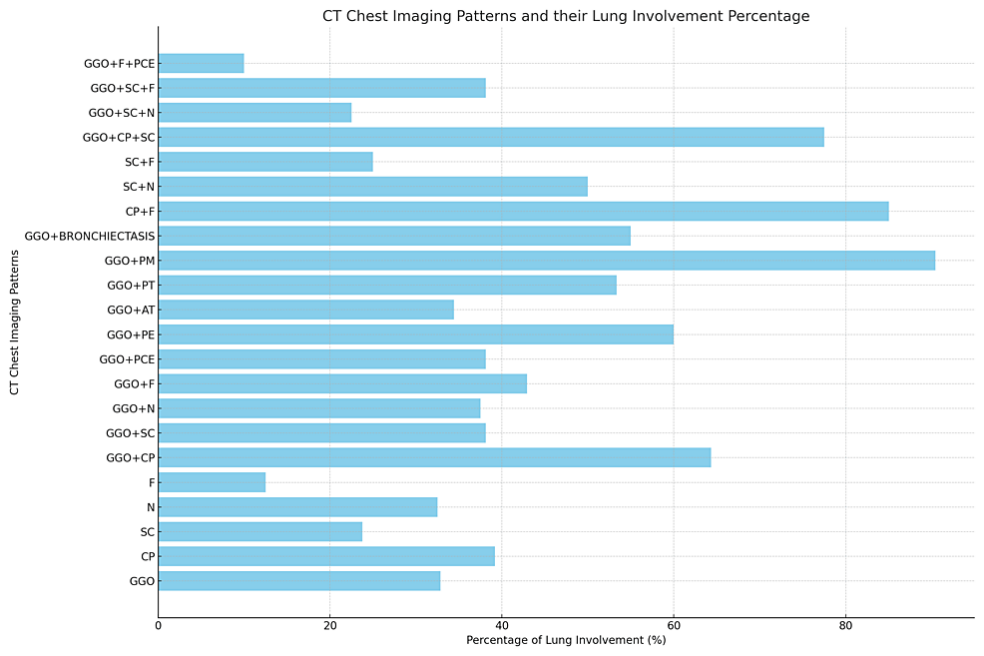
CT chest imaging patterns and their lung involvement percentage LI: lung involvement, GGO: ground glass opacity, CP: crazy paving, SC: subpleural curvilinear, N: nodules, F: fibrosis, CP: crazy paving, SC: subpleural curvilinear, N: nodules, F: fibrosis, PCE: pulmonary consolidation and edema, PE: pulmonary embolism, AT: air trapping, PT: pulmonary tuberculosis, PM: pulmonary metastases, PCE: pulmonary consolidation and edema

**Figure 2:**
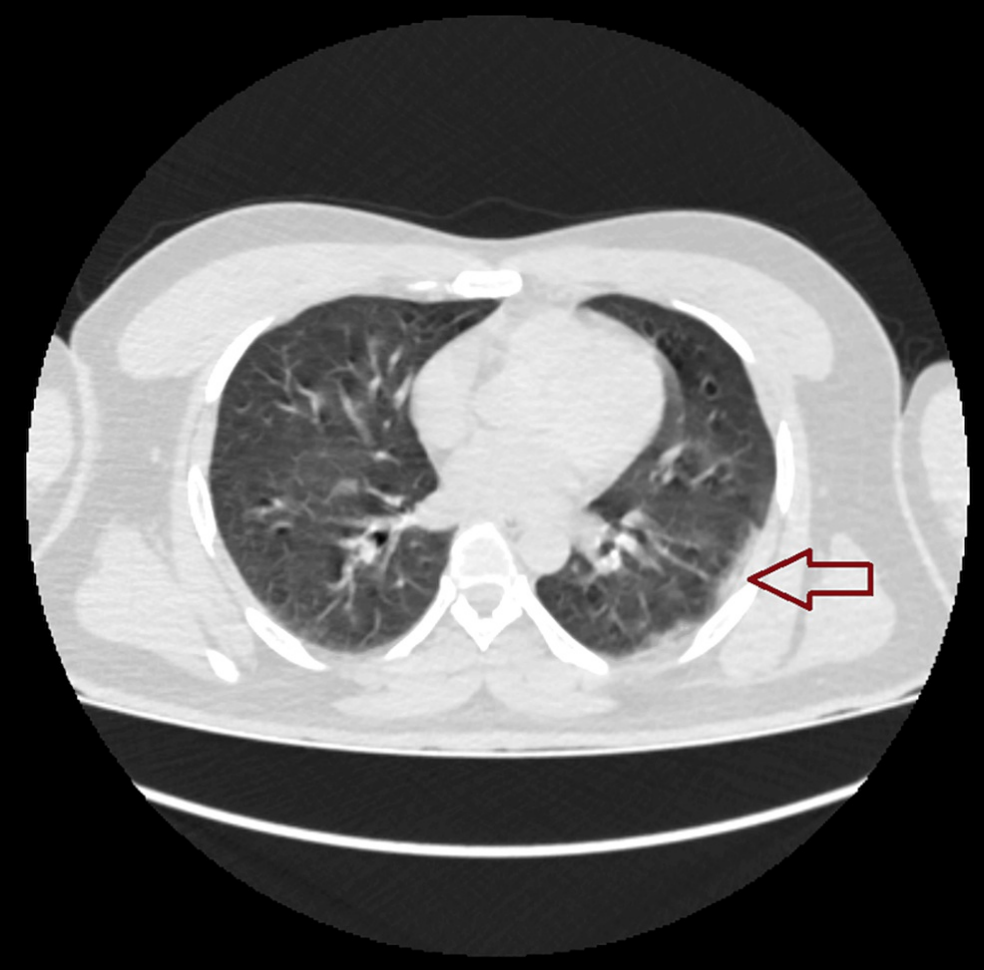
CT chest image showing ground glass opacity

Table 3 and Figure 1 detail the CT chest imaging patterns identified among the patients. Ground-glass opacity (GGO) was the most prevalent pattern, seen in 615 patients, which equated to 32.85% of lung involvement (LI) (Figure 2). Within this subset, 560 patients were discharged with a lung involvement rate of 31.07%, while 55 encountered fatal outcomes, accounting for a lung involvement rate of 50.64%. Patterns that combined GGO with other features, notably pneumomediastinum (GGO+PM), manifested significant mortality rates. Of the six patients diagnosed with the GGO+PM pattern, five succumbed, indicating a lung involvement rate of 96.50%. The (GGO+CP) ground-glass opacity with crazy paving pattern was evident in 19 patients, with an associated lung involvement rate of 64.34%. Among these, seven faced fatal outcomes, representing a lung involvement rate of 72.86%.

**Table 3:** Patterns of lung involvement and outcomes in patients LI: lung involvement, GGO: ground glass opacity, CP: crazy paving, SC: subpleural curvilinear, N: nodules, F: fibrosis, CP: crazy paving, SC: subpleural curvilinear, N: nodules, F: fibrosis, PCE: pulmonary consolidation and edema, PE: pulmonary embolism, AT: air trapping, PT: pulmonary tuberculosis, PM: pulmonary metastases, PCE: pulmonary consolidation and edema

Patterns	Total (LI)	Death (LI)	Discharge (LI)
GGO	615 (32.85%)	55 (50.64%)	560 (31.07%)
CP	3 (39.17%)	1 (77.5%)	2 (20%)
SC	4 (23.75%)	-	4 (23.75%)
N	3 (32.5%)	1 (25%)	2 (36.25%)
F	1 (12.5%)	-	1 (12.5%)
GGO+CP	19 (64.34%)	7 (72.86%)	12 (59.38%)
GGO+SC	76 (38.10%)	4 (66.25%)	72 (36.53%)
GGO+N	4 (37.5%)	-	4 (37.5%)
GGO+F	23 (42.93%)	5 (56.50%)	18 (39.17%)
GGO+PCE	4 (38.13%)	1 (40%)	3 (37.5%)
GGO+PE	3 (60%)	-	3 (60%)
GGO+AT	49 (34.4%)	1 (50%)	48 (34.11%)
GGO+PT	3 (53.33%)	-	3 (53.33%)
GGO+PM	6 (90.42%)	5 (96.50%)	1 (60%)
GGO+BRONCHIECTASIS	1 (55%)	-	1 (55%)
CP+F	1 (85%)	1 (85%)	-
SC+N	1 (50%)	1 (50%)	-
SC+F	1 (25%)	-	1 (25%)
GGO+CP+SC	1 (77.5%)	1 (77.5%)	-
GGO+SC+N	1 (22.5%)	-	1 (22.5%)
GGO+SC+F	4 (38.13%)	-	4 (38.13%)
GGO+F+PCE	1 (10%)	-	1 (10%)

Segmental lung involvement analysis

Right Lung

**Figure 3:**
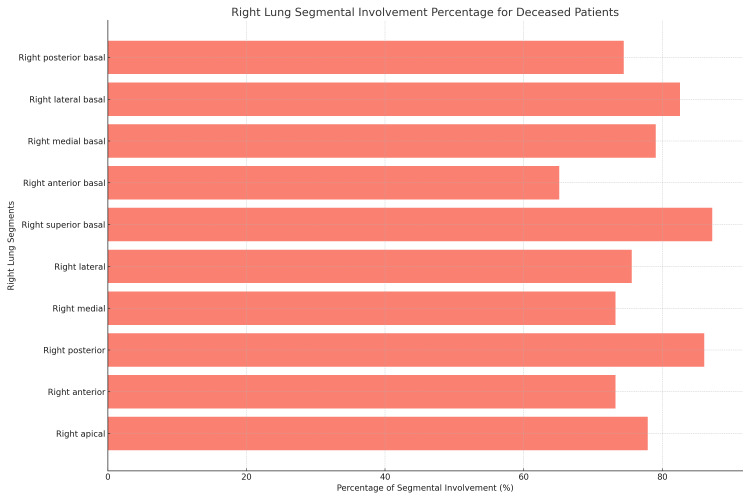
Right lung segmental involvement percentage for deceased patients

**Figure 4:**
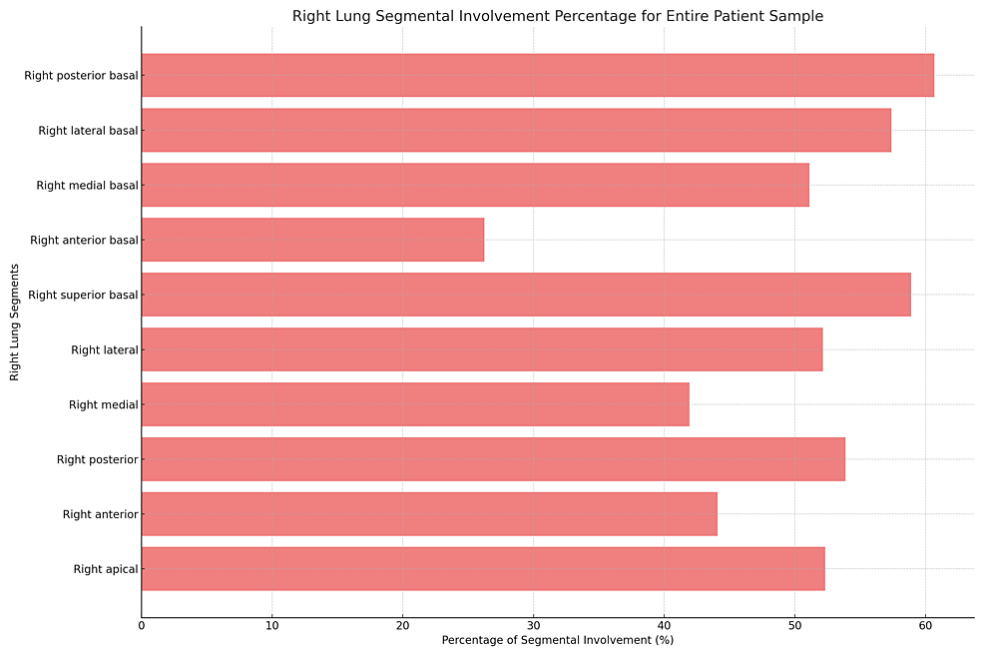
Right lung segmental involvement percentage for the entire patient sample

Referencing Table 4 and Figure 3, among the 86 patients who tragically passed due to the infection, the right posterior segment of the upper lobe displayed involvement in 74 patients (86.04%), and the right superior basal segment exhibited involvement in 75 patients (87.20%). When expanding to the entire patient sample, as shown in Table 5 and Figure 4, the right posterior basal segment recorded the highest involvement, affecting 647 patients (60.69%), followed closely by the right superior basal segment with 628 patients (58.91%).

**Table 4:** Distribution of right lung segment involvement in deceased patients

Segment	Involved (percentage of total)	Not involved (percentage of total)
Right apical	67 (77.90%)	19 (22.09%)
Right anterior	63 (73.25%)	23 (26.74%)
Right posterior	74 (86.04%)	12 (13.95%)
Right medial	63 (73.25%)	23 (26.74%)
Right lateral	65 (75.58%)	21 (24.41%)
Right superior basal	75 (87.20%)	11 (12.79%)
Right anterior basal	56 (65.11%)	30 (34.88%)
Right medial basal	68 (79.06%)	18 (20.93%)
Right lateral basal	71 (82.55%)	15 (17.44%)
Right posterior basal	64 (74.41%)	22 (25.58%)

**Table 5:** Distribution of right lung segments involvement by region

Segment	Involved (percentage of total)	Not involved (percentage of total)
Right apical	558 (52.34%)	508 (47.65%)
Right anterior	470 (44.09%)	596 (55.9%)
Right posterior	574 (53.89%)	491 (46.1%)
Right medial	447 (41.93%)	619 (58.06%)
Right lateral	556 (52.15%)	510 (47.84%)
Right superior basal	628 (58.91%)	438 (41.08%)
Right anterior basal	280 (26.24%)	786 (73.73%)
Right medial basal	545 (51.12%)	521 (48.87%)
Right lateral basal	612 (57.41%)	454 (42.58%)
Right posterior basal	647 (60.69%)	419 (39.3%)

Left Lung

**Figure 5:**
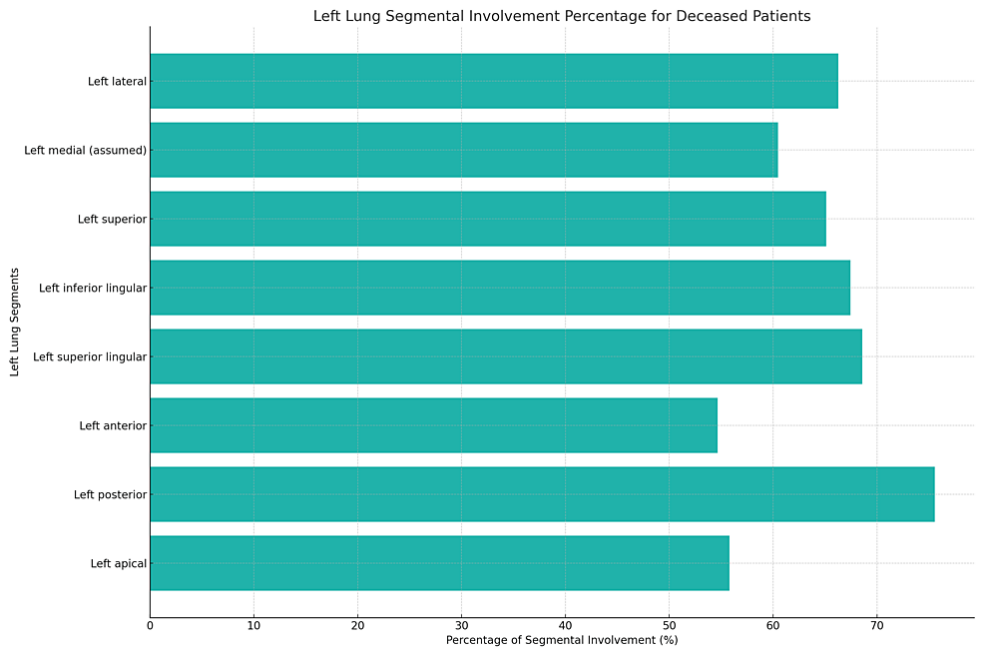
Left lung segmental involvement percentage for deceased patients Left medial, left lateral, left anterior and left posterior being the basal segments

**Figure 6:**
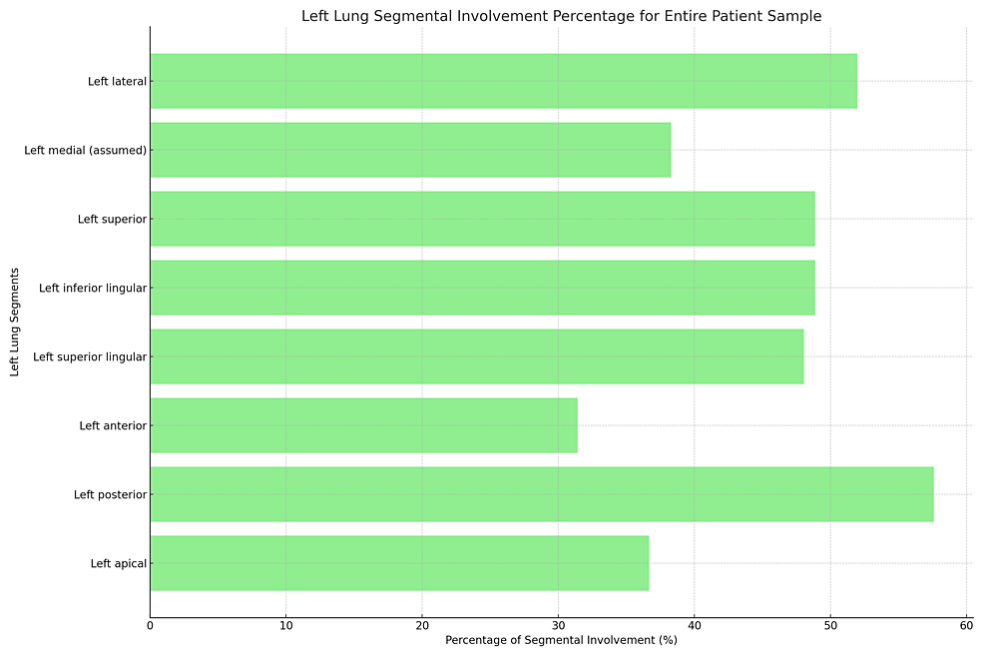
Left lung segmental involvement percentage for the entire patient sample Left medial, left lateral, left anterior and left posterior being the basal segments

As per Table 6 and Figure 5, among deceased patients, the left posterior segment of the upper lobe (part of the apicoposterior segment arbitrarily considered as a separate segment for scoring purposes) showed involvement in 65 individuals (75.58%), and the left superior lingular segment revealed involvement in 59 patients (68.60%) [8,9]. For the broader patient population, Table 7 and Figure 6 indicate that the left posterior segment of the upper lobe was prominently involved, affecting 614 patients (57.59%), while the left lateral segment had involvement in 554 patients (51.97%).

**Table 6:** Distribution of left lung segment involvement in deceased patients

Segment	Involved (percentage of total)	Not involved (percentage of total)
Left apical	48 (55.81%)	38 (44.18%)
Left posterior	57 (66.27%)	29 (33.72%)
Left anterior	47 (54.65%)	39 (45.34%)
Left superior lingular	59 (68.60%)	27 (31.39%)
Left inferior lingular	58 (67.44%)	28 (32.55%)
Left superior basal	56 (65.11%)	30 (34.88%)
Left medial basal (assumed)	52 (60.46%)	34 (39.53%)
Left lateral basal	57 (66.27%)	29 (33.72%)
Left posterior basal	65 (75.58%)	21 (24.41%)
Left anterior basal	44 (51.16%)	42 (48.83%)

**Table 7:** Distribution of left lung segments involvement by region

Segment	Involved (percentage of total)	Not involved (percentage of total)
Left apical	391 (36.67%)	675 (63.32%)
Left posterior	517 (48.49%)	549 (51.5%)
Left anterior	335 (31.42%)	731 (68.57%)
Left superior lingular	512 (48.03%)	554 (51.97%)
Left inferior lingular	522 (48.86%)	544 (51.03%)
Left superior basal	522 (48.86%)	544 (51.03%)
Left medial basal (assumed)	408 (38.27%)	658 (61.72%)
Left lateral basal	554 (51.97%)	512 (48.03%)
Left posterior basal	614 (57.59%)	452 (42.4%)
Left anterior basal	220 (20.63%)	846 (79.36%)

Our findings underscore the increased prevalence of right lung involvement among these patients. The anterior basal segment of the right lung was least affected, with the posterior and lateral basal segments showing more pronounced involvement. On the contrary, in the left lung, the superior and inferior lingular segments manifested significant involvement, contrasting with the minimally affected anterior segment.

A profound correlation was identified between pronounced lung involvement (surpassing 50%) and increased mortality. Specifically, of the 86 fatalities recorded, 41 had more than 50% lung involvement. Furthermore, notable lung involvement was recurrent with the left inferior and superior lingular lobes, as detected in 86.2% (50/58) and 71.1% (42/59) of cases, respectively. This pivotal observation highlights the prognostic significance of lingular segment involvement when evaluating heightened mortality risks.

## Discussion

The SARS-CoV-2 virus, responsible for COVID-19, emerged as a cluster of pneumonia cases in Wuhan, China, in 2019 and quickly spread to other parts of the world. Characteristic clinical symptoms include myalgia, dyspnea, cough, and fever, with primarily human-to-human transmission happening via respiratory droplets [10]. Early diagnosis is crucial in keeping patients isolated and preventing the infection from spreading further [3]. CT scans have emerged as the preferred radiological test for COVID-19. One of the most important factors in the diagnosis of RT PCR-positive patients has been the presence of ground glass opacities, which are frequently present in almost all COVID-19 cases [11,12].

The collected data were analyzed using methods of descriptive analysis and presented in the form of tables. The study found that out of a sample size of 1066, there were 580 men and 486 women studied in the age range of 18-92, with a mean age of 49.3765 and a standard deviation of 14.853. Of the 1066 patients, 242 (22.7%) had no lung involvement, 89 (8.3%) had only right lung involvement, 23 (2.2%) had only left lung involvement, and 712 (66.8%) had both right and left lung involvement. The strong correlation between high lung involvement and higher mortality (41 of 86 deaths had over 50% lung involvement) underscores the need for early and accurate evaluation of lung involvement in COVID-19 patients. While published literature suggests that lung involvement in this condition is primarily restricted to the lower lobes, our findings revealed significant involvement in the upper lobes as well [13]. Additionally, our study found that patients with involvement in the left inferior and superior lingular segments were more likely to have high lung involvement, with 86.2% and 71.1%, respectively, further emphasizing the significance of lingular involvement in predicting higher mortality. Our findings, which indicate that increased lung involvement is associated with higher mortality rates, are consistent with the results reported by Francone et al. [14].

Haseli et al. investigated the distribution of lung involvement in patients with COVID-19 [15]. The findings revealed that the lower lobes of the lungs were more frequently affected, with the right lower lobe showing involvement in 87.3% of cases, while the left lower lobe was involved in 85.7% of cases. Interestingly, the posterior basal segment of the left lower lobe was the most commonly affected area, with a prevalence of 82.5%. The study also analyzed the imaging features of lung involvement and found that peripheral GGO were the most frequent findings, observed in 92.1% of patients. Consolidation was also identified in 42.9% of patients [15]. Intriguingly, our research revealed a correlation between upper lobe involvement and poor prognosis, a finding that is consistent with the study conducted by Baratella et al. [16].

Of the 1066 patients studied, the right upper lobe was involved in 697 patients, the right middle lobe was involved in 599 patients, and the right lower lobe was involved in 651 patients. The left upper lobe was involved in 651 patients, and the left lower lobe was involved in 698 patients. CT chest images of COVID-19 patients revealed various patterns in the lungs indicating active and recent infection, such as ground glass opacities, crazy paving patterns, nodules, foamy, and solid consolidation.

The incidence of various lung patterns, their mean lung involvement, and their clinical outcomes are given in Table 1. GGO (ground glass opacities) had the highest incidence with 615 (57.6%) cases, followed by GGO+CP (ground glass opacities and crazy paving pattern) with 19 (1.78%) cases. GGO+SC (ground glass opacities and solid consolidation) had 76 (7.12%) cases, and GGO+N (ground glass opacities and nodules) had 4 (0.37%) cases. Our study's findings are in accordance with the existing literature on this subject [14,17]. In comparison to an Italian study, our findings indicate lower prevalence percentages of the most common patterns, but with a broader distribution [16,17].

In a study by Barnheim et al., the right and left lower lobes were the most frequently affected regions, with 65% and 63% of patients showing involvement, respectively [18]. Additionally, the right middle lobe and left upper lobe were also significantly involved in 41% and 48% of patients, respectively. Notably, 60% of patients had bilateral lung disease. The study also analyzed the imaging features of lung involvement and found that ground-glass opacities (GGO) were the most common finding, observed in 78% of patients. Consolidation was also identified in 36% of patients, either alone or in combination with GGO. There was a wide range of lobar involvement patterns, with 27% of patients showing disease affecting all five lobes. Only a minority of patients (22%) had no GGO or consolidation on chest CT, while 34% of patients had GGO without consolidation, and 2% had consolidation without GGO [18]. The outcomes of this study demonstrate a level of comparability to those found in the present study.

This study has provided valuable insights into the distribution and patterns of lung involvement found in the CT chests of COVID-19 patients. The study found that both right and left lung involvement was present in 66.8% of the patients, with the right upper lobe being the most commonly affected area. The study also identified various lung patterns, such as ground glass opacities, crazy paving patterns, nodules, foamy, and solid consolidation, among others. These patterns were found to be closely associated with clinical outcomes, with ground glass opacities being the most commonly found pattern and having a higher incidence of death compared to other patterns. The findings of this study are important for the management of COVID-19 patients, as they can help in the early identification of patients at high risk of mortality and the initiation of appropriate treatment [19,20]. The study also highlights the importance of CT chest scans in the diagnosis and management of COVID-19 patients. The results of this study can be used to guide further research and inform the development of clinical guidelines for the management of COVID-19 patients.

This study is confined by its retrospective nature, relying primarily on secondary data analysis, which impedes the determination of causal relationships. The sample is derived exclusively from a singular hospital, potentially narrowing the generalizability of the findings. Further multi-center research is necessitated to validate these results and expand on the lung involvement patterns in diverse populations and settings pertaining to COVID-19.

## Conclusions

This cross-sectional study investigated lung involvement patterns in COVID-19 patients via CT scans and their mortality implications. Ground glass opacities, common in most cases, were significant prognostic markers. Notably, lung involvement exceeding 50% was linked to increased mortality, evident in 41 of the 86 deaths. Additionally, the left inferior and superior lingular lobes showed recurrent involvement in 86.2% and 71.1% of cases, respectively, associated with worse outcomes. Prompt diagnosis, facilitated by CT scans, is crucial for effective COVID-19 management and controlling its spread. Additionally, this study highlights the need for further research into the underlying causes of these lung involvement variations. Such investigations could lead to novel therapies and improved prognostic tools. Overall, this research enhances our understanding of COVID-19 pathogenesis and underscores the diagnostic and prognostic potential of CT scans.

## References

[1] Hao YJ, Wang YL, Wang MY, Zhou L, Shi JY, Cao JM, Wang DP (2022). The origins of COVID-19 pandemic: a brief overview. Transbound Emerg Dis.

[2] Flores-Vega VR, Monroy-Molina JV, Jiménez-Hernández LE, Torres AG, Santos-Preciado JI, Rosales-Reyes R (2022). SARS-CoV-2: Evolution and emergence of new viral variants. Viruses.

[3] (2023). Coronavirus. https://www.who.int/health-topics/coronavirus#tab=tab_1.

[4] Aljondi R, Alghamdi S (2020). Diagnostic value of imaging modalities for COVID-19: scoping review. J Med Internet Res.

[5] Kang Z, Li X, Zhou S (2020). Recommendation of low-dose CT in the detection and management of COVID-2019. Eur Radiol.

[6] Sharif PM, Nematizadeh M, Saghazadeh M, Saghazadeh A, Rezaei N (2022). Computed tomography scan in COVID-19: a systematic review and meta-analysis. Pol J Radiol.

[7] (2024). CDC: COVID-19 and Your Health. https://www.cdc.gov/coronavirus/2019-ncov/symptoms-testing/symptoms.html.

[8] Wasilewski PG, Mruk B, Mazur S, Półtorak-Szymczak G, Sklinda K, Walecki J (2020). COVID-19 severity scoring systems in radiological imaging - a review. Pol J Radiol.

[9] Klein JS, Rosado-de-Christenson ML (2019). A systematic approach to chest radiographic analysis. Diseases of the Chest, Breast, Heart and Vessels 2019-2022: Diagnostic and Interventional Imaging.

[10] Hu B, Guo H, Zhou P, Shi ZL (2021). Characteristics of SARS-CoV-2 and COVID-19. Nat Rev Microbiol.

[11] Hayden GE, Wrenn KW (2009). Chest radiograph vs. computed tomography scan in the evaluation for pneumonia. J Emerg Med.

[12] Simpson S, Kay FU, Abbara S (2020). Radiological Society of North America expert consensus statement on reporting chest CT findings related to COVID-19. Endorsed by the Society of Thoracic Radiology, the American College of Radiology, and RSNA - secondary publication. J Thorac Imaging.

[13] Alam SZ, Muid SA, Akhter A, Rahman AS, Emran MA, Mostakim MTA (2020). HRCT chest evaluation of COVID-19 patients: experience in Combined Military Hospital Dhaka, Bangladesh. J Bangladesh Coll Phys Surg.

[14] Francone M, Iafrate F, Masci GM (2020). Chest CT score in COVID-19 patients: correlation with disease severity and short-term prognosis. Eur Radiol.

[15] Haseli S, Khalili N, Bakhshayeshkaram M, Sanei Taheri M, Moharramzad Y (2020). Lobar distribution of COVID-19 pneumonia based on chest computed tomography findings: a retrospective study. Arch Acad Emerg Med.

[16] Baratella E, Ruaro B, Marrocchio C (2021). Interstitial lung disease at high resolution CT after SARS-CoV-2-related acute respiratory distress syndrome according to pulmonary segmental anatomy. J Clin Med.

[17] Grassi R, Fusco R, Belfiore MP (2020). Coronavirus disease 2019 (COVID-19) in Italy: features on chest computed tomography using a structured report system. Sci Rep.

[18] Bernheim A, Mei X, Huang M (2020). Chest CT findings in coronavirus disease-19 (COVID-19): relationship to duration of infection. Radiology.

[19] Pan F, Yang L, Liang B (2022). Chest CT patterns from diagnosis to 1 year of follow-up in patients with COVID-19. Radiology.

[20] Canan MG, Sokoloski CS, Dias VL (2022). Chest CT as a prognostic tool in COVID-19. Arch Bronconeumol.

